# Comparison of breast cancer risk factors among molecular subtypes: A case‐only study

**DOI:** 10.1002/cam4.2012

**Published:** 2019-02-14

**Authors:** Liwen Zhang, Yubei Huang, Ziwei Feng, Xin Wang, Haixin Li, Fangfang Song, Luyang Liu, Junxian Li, Hong Zheng, Peishan Wang, Fengju Song, Kexin Chen

**Affiliations:** ^1^ Key Laboratory of Breast Cancer Prevention and Therapy in Ministry of Education Key Laboratory of Molecular Cancer Epidemiology of Tianjin Department of Epidemiology and Biostatistics National Clinical Research Center for Cancer Tianjin Medical University Cancer Institute and Hospital Tianjin People's Republic of China; ^2^ Key Laboratory of Breast Cancer Prevention and Therapy in Ministry of Education Department of Cancer Biobank National Clinical Research Centre of Cancer Tianjin Medical University Cancer Institute and Hospital Tianjin People's Republic of China

**Keywords:** breast cancer, epidemiology, molecular subtype, risk factor, TBCCC

## Abstract

Epidemiological studies have a clear definition of the risk factors for breast cancer. However, it is unknown whether the distribution of these factors differs among breast cancer subtypes. We conducted a hospital‐based case‐only study consisting of 8067 breast cancer patients basing on the Tianjin Cohort of Breast Cancer Cases. Major breast cancer subtypes including luminal A, luminal B, human epidermal growth factor receptor 2 (HER2)‐enriched and basal‐like were defined by estrogen receptor, progesterone receptor, HER2, and Ki‐67 status. Variables including demographic characteristics, reproductive factors, lifestyle habits, imaging examination, and clinicopathologic data were collected for patients. Chi‐square test and one‐way analysis of variance were used to compare the distributions of variables among the four breast cancer subtypes. Multivariate logistic regression was used to estimate the odds ratios and associated 95% confidence intervals where luminal A patients served as the reference group. Overall, more commonality rather than heterogeneity on the distributions of factors was found between the four molecular subtypes of breast cancer. The proportion of overweight and obesity were lower in HER2‐enriched subtype. Women with age at menarche ≤13 years were more likely to be found in basal‐like subtype. Postmenopausal women were more frequent in HER2‐enriched and basal‐like subtypes. Women with benign breast disease and higher breast density were more common in HER2‐enriched subtype. Risk factor scoring showed that total risk scores were similar among the four subtypes. HER2‐enriched and basal‐like subtypes were more frequently diagnosed with large tumors. Calcification was more likely to be found in luminal B and HER2‐enriched subtypes, whereas less distributed in basal‐like subtype. Most of the breast cancer risk factors were similarly distributed among the four major breast cancer subtypes; commonality is predominant.

## 1 INTRODUCTION

Breast cancer is the most common malignant tumor and the leading cause of cancer death among women, with an estimated 1.7 million new cases and 521 900 deaths worldwide each year according to the Globocan 2012.^1^ Although China is a relatively low‐incidence country for breast cancer, new cases of breast cancer have been growing at a rate of 3%‐4% per year in recent years, with an incidence of 27.0/100 000 in 2012.^2^, ^3^ Breast cancer is a highly heterogeneous disease. Based on the expression of specific genes, intrinsic subtyping has classified breast cancer into four major subtypes, including luminal A, luminal B, human epidermal growth factor receptor 2 (HER2)‐enriched, and basal‐like breast cancer. Each breast cancer subtype carries distinct clinicpathologic characteristics and prognoses, which may suggest heterogeneous etiologies.^4^

The occurrence of breast cancer is mainly an interaction of genetic factors and environmental factors, and traditional epidemiology studies have made a clear definition of the risk factors for breast cancer. Recent studies showed that established risk factors might have different effect on different intrinsic subtypes, although the results were inconsistent. One study showed increasing body mass index (BMI) significantly reduced the risk of luminal A tumors among premenopausal women, increasing age at menarche was associated with a lower risk of basal‐like subtype.^5^ Another study showed age at first birth was associated with luminal A tumors, and duration of lactation was inversely associated with risk of basal‐like tumors.^6^ In a case‐control study, parity had a protective effect on all subtypes except for basal‐like subtype, breastfeeding was associated with the risk of luminal A, luminal B, and basal‐like subtypes, and increasing age at menarche had a protective effect on luminal A and B subtypes.^7^ More studies are still needed to illustrate the disparity of breast cancer risk factors among subtypes, especially studies from Asian population.

We conducted this study to evaluate the associations between common risk factors and breast cancer subtypes in a Chinese breast cancer cohort, and to summarize the commonality and heterogeneity of breast cancer epidemiological risk factors among breast cancer subtypes.

## 2 METHODS

### 2.1 Study population

This study was a hospital‐based case‐only study basing on the Tianjin Cohort of Breast Cancer Cases (TBCCC). TBCCC is an open prospective cohort study, which was launched since 2004 and aimed to support studies on breast cancer survival, treatment evaluation, disease progression, molecular subtypes, quality of life, and precision medicine among Chinese female breast cancer patients.^8^, ^9^, ^10^, ^11^, ^12^, ^13^ A total of 12 128 newly diagnosed breast cancers patients had been enrolled in TBCCC until March 2014, while an estimated 2000‐3000 breast cancer patients per year will be continuously added to the current open cohort. These newly diagnosed breast cancer patients were defined as patients who were first diagnosed as breast cancer with pathological examination within 6 months after admission in Tianjin Medical University Cancer Institute and Hospital (TJMUCH). All patients were followed up annually with telephone to collect information of recurrence, metastasis, mortality, and further examination and treatment after progression. Hospital information system at TJMUCH was used to confirmed self‐reported information of recurrence, metastasis, and further examination and treatment after admission. Established death registry data in the local region were used to confirmed self‐reported information of mortality. If patients cannot be contacted by telephone, both established cancer registries and death registries were used to ascertain the prognosis of enrolled patients.

All patients in TBCCC must be Chinese residents. All patients were confirmed with pathological examination, and patients without clear pathological examination were excluded. Moreover, patients without written consent and blood samples, or refused to receive baseline survey and further follow‐up were excluded. In this study, male breast cancer patients and patients without molecular subtypes (or cannot be imputed based on relevant test results, detailed information referred to the section “Imputation of breast cancer molecular subtypes”) were also excluded. Finally, a total of 8067 breast cancer patients with complete data were included in this study.

### 2.2 Data collection

Data of demographic characteristics (age, race, marriage, education, occupation, income, insurance, etc.), reproductive factors (age of menarche, menopausal status, age at menopause, pregnancy, living birth, breast feeding, abortion, etc.), lifestyle habits (smoking, alcohol drinking, diet, physical activity, etc.), and body size (height and weight) were investigated by trained physicians with face‐to‐face questionnaire interview. Imaging examination data were recorded on the case report form by sophisticated imaging physicians with at least 5‐year experience on breast imaging diagnosis. Pathology data were collected from the pathological report form recorded by the pathological physicians with at least 3‐year experience on pathological diagnosis.

Risk factors were classified as follows: age at menarche (≤13, 14, 15, ≥16 years), age at first pregnancy (<30, ≥30 years), number of pregnancy (≤1, 2, ≥3), number of live births (≤1, ≥2), months of breastfeeding (≤12, >12) and menopausal status (no, yes), abortion (no, yes), family history (no, yes), benign breast disease (no, yes), hormone replacement therapy (no, yes), oral contraceptive (no, yes), smoking (no, yes), alcohol drinking (no, yes),and negative events (no, yes). BMI is calculated as body weight (kg)/height^2^ (m^2^) and is categorized into four categories: (a) lean (<18.5 kg/m^2^); (b) normal body weight (18.5‐23.9 kg/m^2^); (c) overweight (24.0‐27.9 kg/m^2^); and (d) obese (≥28 kg/m^2^). According to Breast Imaging Reporting and Data System recommended by the American Radiological Society, breast density is divided into four groups as almost entirely fat (<25%), scattered fibroglandular densities (25%‐50%), scattered fibroglandular densities (50%‐75%), and heterogeneously dense (>75%), and also two groups as non‐dense (<50%) and dense (>50%). (Tumor, Node, Metastasis) TNM stages are classified into early stage (0‐IIA) and advanced stage (IIB‐IV). Tumor size was classified into ≤2 cm and >2 cm.

### 2.3 Biomarker detection

Based on gene expression of estrogen receptor (ER), progesterone receptor (PR), HER2, Ki‐67, CK5/6, and epidermal growth factor receptor (EGFR), breast cancer have been classified into four major subtypes: luminal A (ER+ and [or] PR+, Her2−, Ki‐67 < 14%), luminal B (ER+ and [or] PR+, Her2−, Ki‐67 ≥ 14% or ER+ and [or] PR+, Her2+ [luminal Her2]), HER2‐enriched (ER−, PR−, Her2+), and basal‐like (ER−, PR−, Her2−, CK5/6+ and [or] EGFR+). The ER, PR, and HER2 statuses of the patients were extracted from medical records. The results of HER2 were scored semiquantitatively according to the estimated percentage of positively stained tumor cell nuclei and the intensity of nuclear staining (− for no staining, 1+ for weak intensity, 2+ for intermediate intensity, and 3+ for strong intensity). Results of “−” or “1+” were classed as HER2 negative and “2+” or “3+” as positive. According to the ASCO/CAP guide,^14^ positive ER and PR statuses were defined as ≥1% of tumor cells presenting positive nuclear staining. It is worth noting that not all basal‐like breast cancers are triple‐negative breast cancer and not all triple‐negative breast cancers are basal‐like subtype, with an overlap of approximately 70%‐80%.

### 2.4 Imputation of breast cancer molecular subtypes

The molecular subtype for a part of patients was obtained from the pathology report form recorded by pathologists, and the rest was obtained by imputation. A random forest algorithm was used to construct a subtype classifier using the caret R package,^7^ molecular subtypes were predicted based on age, ER, PR, HER2, and Ki‐67. The random forest algorithm is an ensemble or collection of multiple decision tree models. Each tree is grown from a bootstrap sample of the training dataset and each node is split using the best among a randomly selected subset of explanatory variables or features. Forest algorithm injects randomness into the training of the trees, and combines the output of multiple random trees into the final classifier. We split the cases with known molecular subtype into two groups: the training set (n =* *837, known) and the testing set (n* *=* *209, known). Basing on parameters including age, ER, PR, HER2, and Ki‐67, random forest algorithm was used to model and optimize the training set. We validated the testing set using the constructed model and evaluated the performance of the imputation, with an accuracy of more than 99%. Finally, the constructed model was used to impute the cases with unknown molecular subtypes (n =* *7032). After imputation of breast cancer molecular subtypes, there were 4881 luminal A, 1296 luminal B, 1327 HER2‐enriched, and 563 basal‐like breast cancer cases in this study.

### 2.5 Statistical analysis

The measured data and count data were expressed as mean ± SD and n (%), respectively. Chi‐square test and one‐way analysis of variance were used to compare the distributions of demographic characteristics and risk factors among four subtypes of breast cancers. Multivariate logistic regression was used to estimate odds ratios and associated 95% confidence intervals where luminal A patients served as the reference group, since luminal A patients were the most commonly diagnosed breast cancer subtype. Chi‐square test was used to analyze the association of breast cancer subtypes with tumor markers, hormone levels, and clinical features. All statistical tests were two‐sided and *P *<* *0.05 was considered statistically significant. All analyses were performed using the SPSS 23.0 software (SPSS Inc., Chicago, IL, USA).

## 3 RESULTS

For the 8067 breast cancer patients, 60.5% (n =* *4881) were classified as luminal A, 16.1% (n =* *1296) as luminal B, 16.4% (n =* *1327) as HER2‐enriched, and 7.0% (n =* *563) as basal‐like breast cancer. Demographic characteristics of patients by molecular subtypes were summarized in Table 1. Compared with other subtypes, luminal B subtype was more likely to be younger at diagnosis (*P *<* *0.001). There was a statistically difference in marriage status, average monthly income per person, current occupations, and age at first marriage among the four subtypes of breast cancer (*P *<* *0.05). However, no statistical difference was found in education among the four groups (*P *=* *0.771).

**Table 1:** Demographic characteristics of patients by molecular subtypes

Variables	All cases	Luminal A	Luminal B	HER2‐enriched	Basal‐like	*P* value
	(n = 8067, %)	(n* *= 4881, %)	(n* *= 1296, %)	(n* *= 1327, %)	(n* *= 563, %)	
Age						
Mean ± SD (years)	52.3 ± 10.7	52.9 ± 11.0	49.9 ± 9.9	52.4 ± 10.1	52.6 ± 10.8	<0.001
Marriage						
Single	99 (1.2)	61 (1.3)	19 (1.5)	13 (1.0)	6 (1.1)	0.028
Married	7508 (93.6)	4501 (92.9)	1220 (94.5)	1259 (95.1)	528 (94.3)	
Separated/divorce/widowed	414 (5.2)	284 (5.9)	52 (4.0)	52 (3.9)	26 (4.6)	
Education						
No education	423 (6.1)	247 (6.0)	56 (4.9)	87 (7.5)	33 (6.5)	0.771
Primary school	1013 (14.6)	600 (14.5)	165 (14.6)	167 (14.4)	80 (15.8)	
Junior high school	2282 (32.8)	1357 (32.7)	383 (33.7)	380 (32.8)	162 (32.0)	
Senior high school	2309 (33.2)	1392 (33.6)	377 (33.2)	374 (32.3)	166 (32.7)	
Junior college or above	922 (13.3)	552 (13.3)	153 (13.5)	151 (13.0)	66 (13.0)	
Average monthly income per person (RMB)						
<500	262 (3.5)	190 (4.2)	33 (2.7)	26 (2.1)	13 (2.5)	<0.001
500‐999	1100 (14.8)	679 (15.1)	192 (15.9)	153 (12.6)	76 (14.4)	
1000‐1999	3265 (43.8)	1908 (42.4)	539 (44.6)	580 (47.6)	238 (45.1)	
2000‐2999	1801 (24.2)	1082 (24.1)	296 (24.5)	283 (23.2)	140 (26.5)	
≥3000	1024 (13.7)	639 (14.2)	148 (12.3)	176 (14.4)	61 (11.6)	
Current occupations						
Yes	3781 (48.3)	2194 (46.5)	690 (54.5)	634 (48.8)	263 (47.7)	<0.001
No	4055 (51.7)	2526 (53.5)	576 (45.5)	665 (51.2)	288 (52.3)	
Age at first marriage (years)						
<30	7554 (95.3)	4546 (94.8)	1221 (96.2)	1248 (95.2)	539 (97.1)	0.029
≥30	376 (4.7)	249 (5.2)	48 (3.8)	63 (4.8)	16 (2.9)	

HER2, human epidermal growth factor receptor 2.

As shown in Table 2, compared with women with age at menarche ≥16 years old, the proportion of women with age at menarche ≤13 years old among basal‐like cancers (29.1%) was significantly higher than that among luminal A cancers (26.7%). Compared with premenopause women, the proportions of postmenopause women among either basal‐like cancers (60.0%) or HER2‐enriched cancers (62.6%) were significantly higher than that among luminal A cancers (53.4%, both *P *<* *0.05). Moreover, overweight (36.9% vs 38.7%) and obesity (15.0% vs 18.8%) were significantly less distributed in HER2 subtype (both *P *<* *0.05). Women with benign breast disease were significantly more common in HER2‐enriched subtype (39.4% vs 35.8%, *P *<* *0.05). Dense breast women were significantly associated with HER2 subtype (49.3% vs 45.1%, *P *<* *0.05).

**Table 2:** Relationship between risk factors and breast cancer subtypes^a^

	Luminal A	Luminal B		HER2‐enriched		Basal‐like	
	n (%)	n (%)	OR (95% CI)	n (%)	OR (95% CI)	n (%)	OR (95% CI)
BMI							
<18.5	94 (1.9)	28 (2.2)	1.31 (0.78‐2.21)	25 (1.9)	0.98 (0.56‐1.73)	14 (2.5)	1.89 (0.98‐3.64)
18.5‐23.9	1969 (40.6)	582 (45.1)	1.00 (ref)	611 (46.1)	1.00 (ref)	225 (40.3)	1.00 (ref)
24.0‐27.9	1878 (38.7)	479 (37.1)	1.00 (0.85‐1.18)	489 (36.9)	0.77 (0.65‐0.91)^a^	224 (40.1)	1.02 (0.81‐1.28)
≥28	910 (18.8)	202 (15.6)	0.83 (0.67‐1.03)	199 (15.0)	0.67 (0.54‐0.83)^a^	96 (17.2)	0.93 (0.69‐1.25)
Age of menarche (years)							
≤13	1296 (26.7)	377 (29.3)	1.16 (0.96‐1.41)	371 (28.1)	1.17 (0.96‐1.42)	163 (29.1)	1.33 (1.02‐1.74)^a^
14	1072 (22.1)	275 (21.4)	1.04 (0.85‐1.27)	255 (19.3)	0.99 (0.81‐1.22)	120 (21.4)	1.08 (0.82‐1.44)
15	779 (16.1)	202 (15.7)	1.02 (0.82‐1.26)	219 (16.6)	1.12 (0.90‐1.38)	83 (14.8)	0.98 (0.72‐1.35)
≥16	1701 (35.1)	432 (33.6)	1.00 (ref)	477 (36.1)	1.00 (ref)	195 (34.8)	1.00 (ref)
Number of pregnancy							
≤1	796 (16.5)	226 (17.6)	0.99 (0.68‐1.43)	226 (17.1)	1.03 (0.72‐1.48)	91 (16.3)	1.13 (0.68‐1.86)
2	1368 (28.3)	386 (30.0)	1.02 (0.85‐1.22)	394 (29.8)	1.04 (0.86‐1.25)	171 (30.6)	1.11 (0.86‐1.44)
≥3	2665 (55.2)	674 (52.4)	1.00 (ref)	701 (53.1)	1.00 (ref)	296 (53.0)	1.00 (ref)
Age at first full‐term pregnancy (years)							
<30	4166 (89.6)	1131 (91.7)	1.00 (ref)	1158 (90.6)	1.00 (ref)	491 (91.3)	1.00 (ref)
≥30	481 (10.4)	103 (8.3)	1.15 (0.82‐1.61)	120 (9.4)	0.76 (0.53‐1.10)	47 (8.7)	1.27 (0.81‐1.98)
Number of live births							
≤1	3083 (63.9)	868 (67.6)	0.89 (0.71‐1.11)	860 (65.2)	0.98 (0.78‐1.22)	345 (61.7)	0.81 (0.59‐1.11)
≥2	1740 (36.1)	416 (32.4)	1.00 (ref)	459 (34.8)	1.00 (ref)	214 (38.3)	1.00 (ref)
Months of breast feeding							
≤12	1855 (40.7)	502 (41.4)	0.98 (0.82‐1.16)	481 (39.1)	0.91 (0.77‐1.09)	200 (38.0)	0.90 (0.70‐1.15)
>12	2700 (59.3)	711 (58.6)	1.00 (ref)	749 (60.9)	1.00 (ref)	327 (62.0)	1.00 (ref)
Abortion							
No	1353 (28.2)	350 (27.3)	1.00 (ref)	373 (28.4)	1.00 (ref)	171 (30.8)	1.00 (ref)
Yes	3449 (71.8)	931 (72.7)	1.00 (0.77‐1.31)	939 (71.6)	0.93 (0.72‐1.20)	385 (69.2)	0.92 (0.65‐1.30)
Oral contraceptive							
No	3956 (85.6)	1083 (87.1)	1.00 (ref)	1101 (85.7)	1.00 (ref)	472 (86.3)	1.00 (ref)
Yes	667 (14.4)	161 (12.9)	0.97 (0.78‐1.20)	183 (14.3)	0.98 (0.79‐1.20)	75 (13.7)	0.94 (0.70‐1.27)
HRT							
No	4375 (94.6)	1188 (95.2)	1.00 (ref)	1204 (93.6)	1.00 (ref)	522 (95.1)	1.00 (ref)
Yes	250 (5.4)	60 (4.8)	1.00 (0.70‐1.43)	82 (6.4)	1.29 (0.94‐1.77)	27 (4.9)	0.92 (0.56‐1.52)
Menopause							
No	2245 (46.6)	674 (52.6)	1.00 (ref)	490 (37.4)	1.00 (ref)	221 (40.0)	1.00 (ref)
Yes	2572 (53.4)	608 (47.4)	1.15 (0.91‐1.45)	820 (62.6)	1.94 (1.53‐2.46)^a^	332 (60.0)	1.89 (1.36‐2.62)^a^
Benign breast disease							
No	3056 (64.2)	763 (60.0)	1.00 (ref)	791 (60.6)	1.00 (ref)	352 (63.7)	1.00 (ref)
Yes	1705 (35.8)	508 (40.0)	1.09 (0.93‐1.27)	514 (39.4)	1.24 (1.06‐1.45)^a^	201 (36.3)	1.05 (0.84‐1.31)
Smoking							
No	4268 (89.4)	1152 (89.9)	1.00 (ref)	1179 (89.5)	1.00 (ref)	510 (90.9)	1.00 (ref)
Yes	507 (10.6)	130 (10.1)	1.08 (0.85‐1.37)	138 (10.5)	0.97 (0.76‐1.23)	51 (9.1)	0.77 (0.53‐1.10)
Alcohol drinking							
No	4659 (97.9)	1249 (97.6)	1.00 (ref)	1292 (98.3)	1.00 (ref)	549 (98.0)	1.00 (ref)
Yes	101 (2.1)	31 (2.4)	1.09 (0.66‐1.79)	23 (1.7)	0.73 (0.40‐1.32)	11 (2.0)	1.05 (0.50‐2.23)
Negative events							
No	3150 (67.6)	868 (69.1)	1.00 (ref)	886 (68.9)	1.00 (ref)	368 (67.4)	1.00 (ref)
Yes	1507 (32.4)	389 (30.9)	0.98 (0.83‐1.15)	400 (31.1)	0.92 (0.79‐1.08)	178 (32.6)	1.00 (0.80‐1.24)
First‐degree family history of breast cancer							
No	4540 (93.9)	1223 (94.7)	1.00 (ref)	1240 (94.0)	1.00 (ref)	530 (94.8)	1.00 (ref)
Yes	296 (6.1)	69 (5.3)	0.90 (0.65‐1.25)	79 (6.0)	0.97 (0.71‐1.33)	29 (5.2)	1.02 (0.66‐1.56)
Breast density							
Non‐dense	1585 (54.9)	468 (48.9)	1.00 (ref)	578 (50.7)	1.00 (ref)	247 (56.4)	1.00 (ref)
Dense	1304 (45.1)	490 (51.1)	0.95 (0.77‐1.18)	563 (49.3)	1.42 (1.16‐1.73)^a^	191 (43.6)	0.92 (0.69‐1.23)

HER2, human epidermal growth factor receptor 2; BMI, body mass index; HRT, hormone replacement therapy.

Odds ratios were adjusted for age, marriage, education, average monthly income, current occupations, age at first marriage, and all above risk factors for breast cancer.

a Statistically significant at *P *<* *0.05.

**Figure 1:**
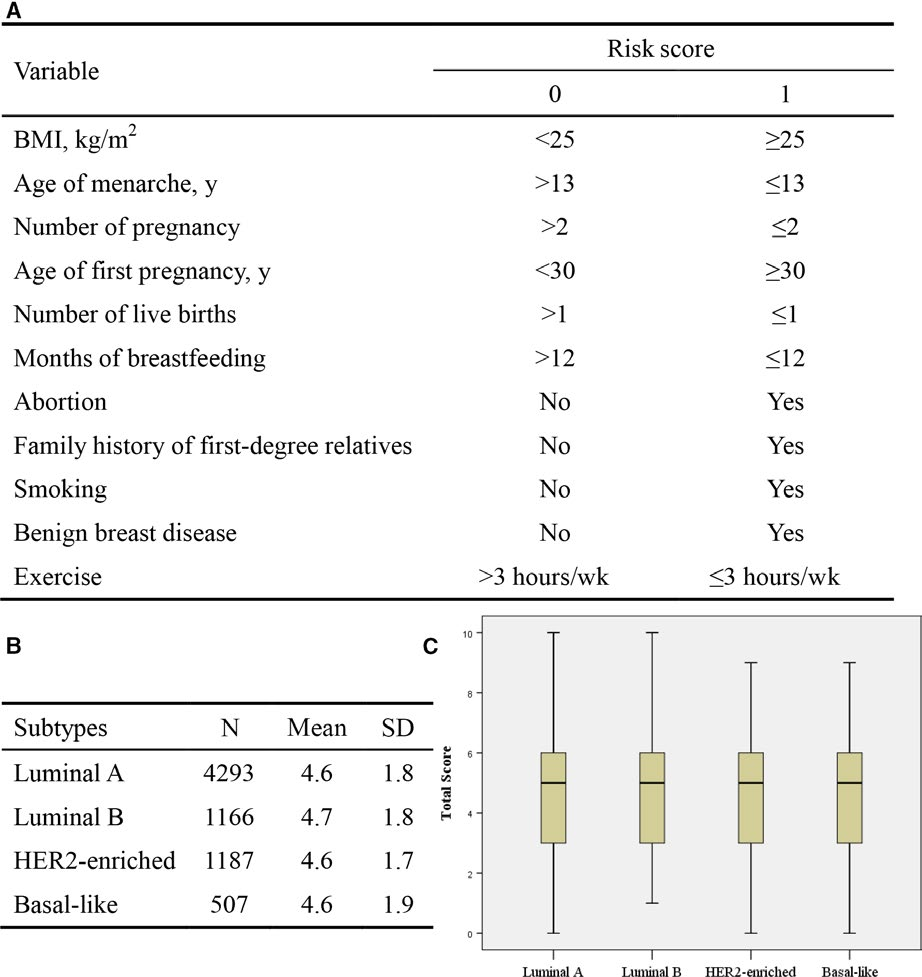
Risk factors scoring. (A) Risk scoring system, each variable was divided into two categories, with 0 representing lower risk for breast cancer and 1 representing higher breast cancer risk (B) Average scores for the four subtypes (C) Total risk scores for the four subtypes were presented in a box plot

Furthermore, we constructed a risk scoring system including a total of 11 variables for to summarize breast cancer risk for each patient. Each variable was divided into two categories, with 0 representing lower risk for breast cancer and 1 representing higher breast cancer risk (Figure 1). We added the above 11 risk factors together and calculated a total risk score for each patient. The average scores for four subtypes were 4.6, 4.7, 4.6, and 4.6, respectively. Box plot showed that total risk scores for the four molecular subtypes were similar (Figure 1).

**Figure 2:**
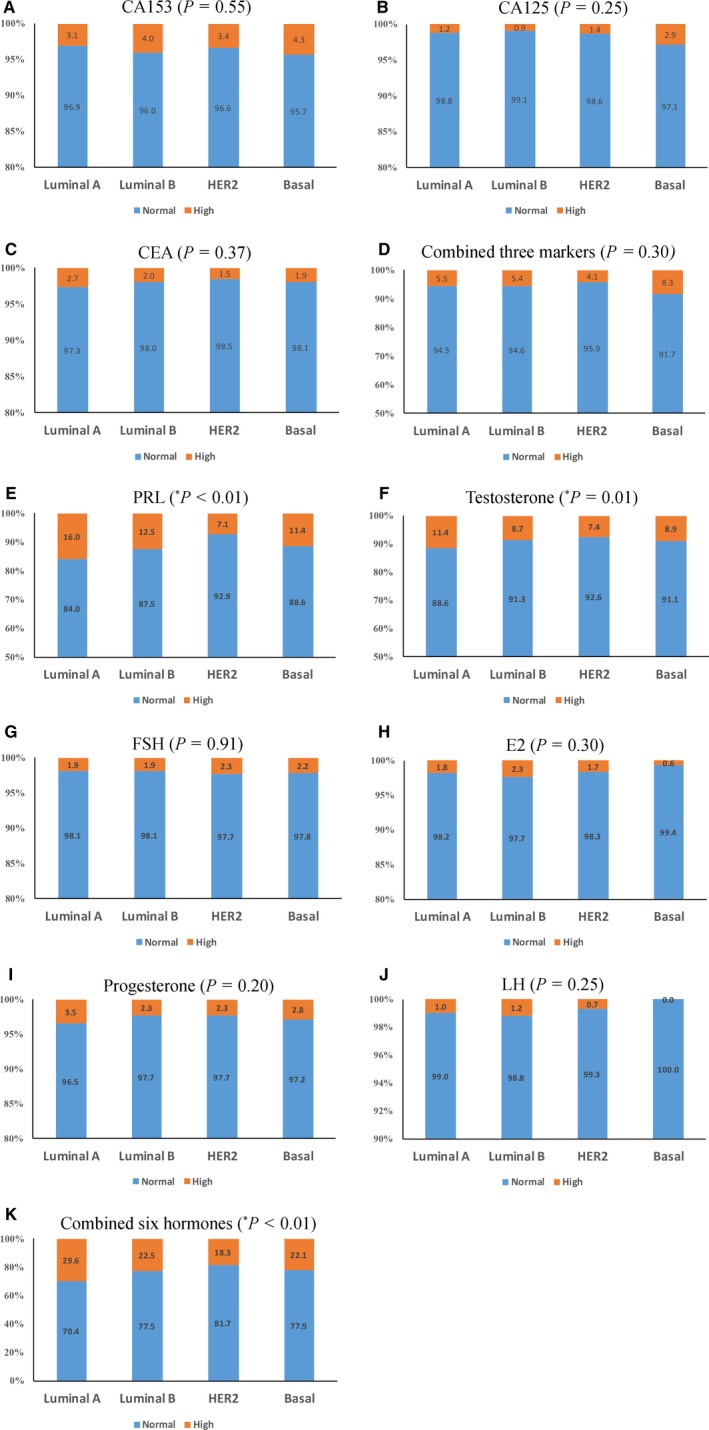
Tumor markers, hormone levels, and breast cancer subtypes. (A) CA153, (B) CA125, (C) CEA, and (D) combined three markers; (E) prolactin (PRL), (F) testosterone, (G) follicle‐stimulating hormone (FSH), (H) estradiol (E2), (I) progesterone, (J) luteinizing hormone (LH), and (K) combined six hormones

Of the 8067 cases, CA153, CA125, and Carcinoembryonic Antigen (CEA) were measured in 4803, 2047, and 3270 breast cancer patients, respectively. There was no statistically significant difference in the proportion of CA153 (*P *=* *0.55), CA125 (*P *=* *0.25), and CEA (*P *=* *0.37) beyond the reference range in different subtypes (Figure 2). CA125, CA153, and CEA were measured simultaneously in 1867 breast cancer patients and no significant difference was found for the three markers combined among the four subtypes (χ^2^ = 3.653, *P *=* *0.30). Prolactin (PRL) and testosterone were measured in 5000 and 5786 breast cancer patients, with 15.7% and 10.7% of patients being above or below the reference range, respectively. The abnormal proportions of PRL and testosterone in different subtypes of breast cancer were statistically significant (*P *<* *0.01), whereas other four hormones as follicle‐stimulating hormone, estradiol (E2), progesterone, and luteinizing hormone were not significantly different among the four subtypes (*P *>* *0.05) (Figure 2).

The tumor characteristics of patients by molecular subtypes were shown in Table 3. The proportion of calcification is highest in luminal B (73.3%) and lowest in basal‐like (53.6%) subtype. Luminal A cases were more likely to have American Joint Committee on Cancer (AJCC) early stage (65.9%) and tumor size ≤2 cm (50.7%). Compared to luminal A cases, calcification was associated with an increased odds of luminal B and HER2‐enriched subtype, whereas with a lower odds of basal‐like subtype. HER2‐enriched and basal‐like breast cancers were more often to have large tumors. There was no significant association between molecular subtype and lymph node metastasis (*P *=* *0.114).

**Table 3:** Tumor characteristics of patients by molecular subtypes

	Luminal A	Luminal B		HER2‐enriched		Basal‐like	
	n (%)	n (%)	OR (95% CI)	n (%)	OR (95% CI)	n (%)	OR (95% CI)
Calcification							
No	1704 (36.0)	342 (26.7)	1.00 (ref)	371 (28.6)	1.00 (ref)	256 (46.4)	1.00 (ref)
Yes	3035 (64.0)	937 (73.3)	1.44 (1.22‐1.70)^*^	926 (71.4)	1.33 (1.13‐1.56)^*^	296 (53.6)	0.69 (0.55‐0.86)^*^
Tumor stage							
Stage 0‐IIA	2246 (65.9)	580 (59.1)	1.00 (ref)	591 (61.3)	1.00 (ref)	238 (63.8)	1.00 (ref)
Stage IIB‐IV	1163 (34.1)	402 (40.9)	1.28 (0.95‐1.71)	373 (38.7)	1.22 (0.90‐1.64)	135 (36.2)	1.06 (0.66‐1.69)
Tumor size (cm)							
≤2	2221 (50.7)	509 (42.5)	1.00 (ref)	521 (43.5)	1.00 (ref)	185 (38.9)	1.00 (ref)
>2	2158 (49.3)	690 (57.5)	1.19 (0.99‐1.42)	676 (56.5)	1.28 (1.07‐1.53)^*^	291 (61.1)	1.65 (1.27‐2.15)^*^
Lymph node metastasis							
No	1865 (51.2)	493 (47.3)	1.00 (ref)	517 (50.6)	1.00 (ref)	213 (53.1)	1.00 (ref)
Yes	1781 (48.8)	549 (52.7)	0.92 (0.72‐1.19)	505 (49.4)	0.80 (0.62‐1.04)	188 (46.9)	0.72 (0.48‐1.09)

HER2, human epidermal growth factor receptor 2.

Statistically significant at *P *<* *0.05.

## 4 DISCUSSION

In this hospital‐based case‐only study, more commonality rather than heterogeneity on the distributions of factors was found between the four molecular subtypes of breast cancer. The differences between four molecular subtypes of breast cancers are mainly manifested in clinical characteristics such as calcification, stage, tumor size, and mammary gland‐related hormone levels. Overall, risk factor scoring indicated that total risk scores for the four molecular subtypes were similar.

Many studies have evaluated the association between breast cancer risk factors and breast cancer subtypes. In a case‐control study in East Asian women, they found overweight, late menopause, and lack of breastfeeding appear to increase risk of both luminal and ER‐PR tumors.^15^ In a cross‐sectional study of 7020 patients, Brouckaert et al. found BMI was linearly related to the probabilities of luminal B and HER2‐like breast cancer subtypes.^16^ Phipps et al. found breast density was similarly positively associated with risk of all subtypes, BMI was positively associated with risks of ER‐positive and triple‐negative breast cancer.^17^ Au et al. suggest a correlation of the occurrence of luminal‐like BC subtypes with low parity and short or no duration of breastfeeding.^18^ In a nested case‐control study, number of pregnancies was inversely associated with relative risk of luminal‐like breast cancers, hormone therapy use was strongly associated with risk of luminal‐like breast cancer.^19^ In a study of reproductive factors and risk of triple‐negative breast cancer, breastfeeding decreases the risk of TNBC.^20^ These researches are similar to our findings that a few factors were differently associated with certain subtypes, but in general there is no substantial difference. Researchers may emphasize the special risk factors for special subtypes, while these factors cannot be well replicated in other studies, the disparity may be caused simply by chance, but the underlying biological role of certain factors should not be overlooked and need further research.

In this study, women with menarche age ≤13 years were more likely to be found in basal‐like subtype. In a meta‐analysis of 12 populations by Yang, they found women with menarche age <12 years increased 1.16 times the risk of ER‐positive tumors.^21^ Ma et al. showed that late menarche age can reduce the risk of all subtypes breast cancer.^22^ Our study found postmenopausal women were more frequent in HER2‐enriched and basal‐like subtypes. In a population‐based case‐case study consisting of 2710 women, they found that age at menopause were positively associated with odds of triple‐negative breast cancer.^23^ In a case‐control study in Southeast Asia, late age of menopause was associated with an increased risk of luminal and basal‐like tumors.^15^ Women with benign breast disease and higher breast density were more common in HER2‐enriched subtype, whereas Holm et al. did not find a significant difference between benign breast disease and breast cancer subtypes.^7^ The association between breast density and breast cancer subtypes is still uncertain.^17^, ^24^, ^25^ In our study, reproductive factors such as number of pregnancy, number of live births and breastfeeding have no difference among the four subtypes. Current findings on reproductive factors are inconsistent.^20^, ^22^, ^26^, ^27^, ^28^ These findings require confirmation in other studies, and further researches are needed to establish the association between factors and breast cancer subtypes.

The differences between different breast cancer molecular subtypes are mainly manifested in tumor characteristics. In this study, we found serum CA153, CEA, and CA125 were not statistically different between the four groups. Similar to our results, Moazzey et al. reported that CA153 and CEA were not significantly different among different subgroups.^29^ We found that hormones such as PRL and testosterone were significantly different in different subtypes of breast cancer. Similar to our findings, Hachim et al. found PRLR expression was highest in the luminal A subtype,^30^ Guo et al. found a testosterone increased the risk of ER+ breast cancer.^31^ Furthermore, Cen et al. found that calcification is associated with luminal A and HER2‐enriched subtypes,^32^ in consistent with our results. In our study, there was a significant difference in tumor stage and tumor size. In a retrospective study of Chinese women,^33^ they found the differences between tumor size, lymph node metastasis, AJCC tumor stage, and molecular subtypes. HER2‐enriched breast cancer has higher lymph node metastasis and higher AJCC tumor stage.

Although our study benefits from a large sample size, comprehensively collected data on a large number of breast cancer risk factors and clinical factors, as well as imaging examination data, several limitations must be acknowledged. First, our study was not designed as a case‐control study, which made it difficult to quantify the exact risk for the development of breast cancer subtypes. However, some literature evaluated differences among breast cancer subtypes through case‐case studies, like our study.^34^, ^35^, ^36^ Second, we only had a small part of subtype information from the pathological report form recorded by the pathological physicians and predicted subtype for the rest. It is regrettable that we cannot get replication from publicly available database such as The Cancer Genome Atlas (TCGA) due to incomplete information on the necessary parameters. However, the use of this subtype classifier may have improved accuracy compared with a previously used Immunohistochemistry (IHC)‐based method. Further validation is warranted.

## 5 CONCLUSION

In conclusion, most of breast cancer risk factors and tumor markers for different subtypes of breast cancer are similar, except a few factors for certain subtypes, and the difference is not substantial. The differences between different breast cancer molecular subtypes are mainly manifested in tumor characteristics such as calcification, stage, tumor size, and mammary gland‐related hormone levels, etc. The molecular classification of breast cancer is of great significance in guiding clinical work.

## CONFLICT OF INTEREST

The authors declare that they have no competing interests.
